# Emergence of E484K Mutation Following Bamlanivimab Monotherapy among High-Risk Patients Infected with the Alpha Variant of SARS-CoV-2

**DOI:** 10.3390/v13081642

**Published:** 2021-08-19

**Authors:** Nathan Peiffer-Smadja, Antoine Bridier-Nahmias, Valentine Marie Ferré, Charlotte Charpentier, Mathilde Garé, Christophe Rioux, Aude Allemand, Philippa Lavallée, Jade Ghosn, Laura Kramer, Diane Descamps, Yazdan Yazdanpanah, Benoit Visseaux

**Affiliations:** 1 IAME, Inserm, Université de Paris, UMR1137, 75018 Paris, France; antoine.bridier-nahmias@inserm.fr (A.B.-N.); valentinemarie.ferre@aphp.fr (V.M.F.); charlotte.charpentier@aphp.fr (C.C.); jade.ghosn@aphp.fr (J.G.); diane.descamps@aphp.fr (D.D.); yazdan.yazdanpanah@aphp.fr (Y.Y.); benoit.visseaux@aphp.fr (B.V.); 2 Service des Maladies Infectieuses et Tropicales, Hôpital Bichat Claude Bernard, AP-HP, 75018 Paris, France; mathilde.gare@aphp.fr (M.G.); christophe.rioux@aphp.fr (C.R.); aude.allemand@aphp.fr (A.A.); 3 Health Protection Research Unit in Healthcare Associated Infections and Antimicrobial Resistance, National Institute for Health Research, Imperial College London, London SW7 2AZ, UK; 4 Service de Virologie, Hôpital Bichat Claude Bernard, AP-HP, 75018 Paris, France; 5 Department of Neurology and Stroke Center, Hôpital Bichat Claude Bernard, AP-HP, 75018 Paris, France; philippa.lavallee@aphp.fr; 6 Pharmacy Department, Hôpital Bichat Claude Bernard, AP-HP, 75018 Paris, France; laura.kramer@aphp.fr

**Keywords:** monoclonal antibodies, SARS-CoV-2, COVID-19, variants, resistance

## Abstract

An Emergency Use Authorization was issued in the United States and in Europe for a monoclonal antibody monotherapy to prevent severe COVID-19 in high-risk patients. This study aimed to assess the risk of emergence of mutations following treatment with a single monoclonal antibody. Bamlanivimab was administered at a single dose of 700 mg in a one-hour IV injection in a referral center for the management of COVID-19 in France. Patients were closely monitored clinically and virologically with nasopharyngeal RT-PCR and viral whole genome sequencing. Six patients were treated for a nosocomial SARS-CoV-2 infection, all males, with a median age of 65 years and multiple comorbidities. All patients were infected with a B.1.1.7 variant, which was the most frequent variant in France at the time, and no patients had E484 mutations at baseline. Bamlanivimab was infused in the six patients within 4 days of the COVID-19 diagnosis. Four patients had a favorable outcome, one died of complications unrelated to COVID-19 or bamlanivimab, and one kidney transplant patient treated with belatacept died from severe COVID-19 more than 40 days after bamlanivimab administration. Virologically, four patients cleared nasopharyngeal viral shedding within one month after infusion, while two presented prolonged viral excretion for more than 40 days. The emergence of E484K mutants was observed in five out of six patients, and the last patient presented a Q496R mutation potentially associated with resistance. CONCLUSIONS: These results show a high risk of emergence of resistance mutants in COVID-19 patients treated with monoclonal antibody monotherapy.

## 1. Introduction

Preliminary data available in late 2020 has led several countries to give an emergency approval for the use of a monoclonal antibody monotherapy in the early phase of COVID-19 in high-risk patients [1]. In an interim analysis from a phase two randomized, double-blind, placebo-controlled clinical trial involving 452 outpatients with recently diagnosed mild or moderate COVID-19, monoclonal anti-Spike antibody bamlanivimab appeared to accelerate the decline in viral load and provided encouraging results regarding clinical outcomes and COVID-19 related hospitalizations [2,3]. Following these and other results, an Emergency Use Authorization (EUA) to use bamlanivimab against SARS-CoV-2 was issued in November 2020 by the FDA for the early treatment (within 10 days of symptoms onset) of high-risk patients [4]. In March 2021, the European Medicines Agency (EMA) considered that, despite uncertainties regarding the benefits of monoclonal antibody monotherapies, bamlanivimab alone could be used as a treatment option in high-risk patients [5]. In France, a Temporary Use Authorization (TUA) was issued on 27 February 2021 for the use of bamlanivimab, within 5 days from symptom onset, to treat adults with a positive PCR for SARS-CoV-2 and mild to moderate COVID-19 symptoms and at high-risk of severe COVID-19. Eligible patients under the TUA were patients over 80 or under 80 years on dialysis or immunocompromised (solid organ transplant, chemotherapy, etc.) [6]. The most important drawback counterbalancing the individual benefits of bamlanivimab is the risk of mutation emergence, inducing possible resistance to a single monoclonal anti-Spike antibody. Several case reports have demonstrated such selection, especially the E484K mutation, in patients treated with convalescent plasma or monoclonal antibody therapy [7,8]. Moreover, bamlanivimab demonstrated a strongly decreased in vitro neutralization activity against SARS-CoV-2 variants harboring the E484K mutation [9,10].

As the potential individual benefit of these treatments in high-risk patients was considered greater than the risk of mutant selection, bamlanivimab monotherapy was used in France for a few weeks before the availability of combinations of monoclonal antibodies. In this work, we describe the clinical and virological follow-up of the six patients who received the single monoclonal anti-Spike antibody bamlanivimab in our center.

## 2. Methods

### 2.1. Patients and Samples

Bamlanivimab was administered at a single dose of 700 mg in a one-hour IV injection in Bichat-Claude Bernard Hospital, a referral center for the management of COVID-19 in Paris, France. All patients agreed to receive the treatment as part of the TUA. Clinical and virological data were prospectively recorded over an 8-week period. Iterative SARS-CoV-2 nasopharyngeal PCR was carried out at Bamlanivimab initiation, at day 7 in the case of an absence of viral load decrease, with screening of variants of concerns (VOC) and viral whole-genome sequencing, and thereafter upon viral load kinetic. The research was approved by the local ethics committee, No. CER-2021-75.

### 2.2. SARS-CoV-2 RT-PCR Detection

Nasopharyngeal samples were tested by RT-PCR using either the Cobas^®^ SARS-CoV-2 (Roche, Switzerland) on a Cobas^®^ 6800 system [11] or the NeuMoDx^®^ SARS-CoV-2 Assay (QIAgen, Germany) on a NeuMoDx^®^ 288 system [12]. Cycle threshold (Ct) values of the ORF1ab and N targets were used as a proxy for viral load for the Cobas and the NeuMoDx assay, respectively. All Ct values above 40 were considered negative for all tests, as recommended by the respective manufacturers. Screening for SARS-CoV-2 variants of concern (VOC) was performed using real-time specific RT-PCR SARS-CoV-2 Spike (i.e., deletion 69-70, E484K and N501Y mutations) (TIB Molbiol, Berlin, Germany).

### 2.3. Viral Whole-Genome Sequencing

Full SARS-CoV-2 genome sequencing was conducted from nasopharyngeal primary clinical samples. Reverse transcription was performed with SuperScript IV with random hexamers after acid nucleic extraction using the MagNA Pure LC Total Nucleic Acid Isolation Kit (Roche, Basel, Switzerland). Tiling PCR amplification was performed according to the Artic protocol (nCoV-2019 sequencing protocol v2) with two pools of primers (ARTIC nCoV-2019 V3 panel) [13]. Libraries were prepared with NEBNext Companion Module for Oxford Nanopore Technologies, Ligation Sequencing (SQK-LSK 109), and sequenced using MinION R9.4.1 flow cells. All sequences obtained were deposited in GISAID.

### 2.4. Viral Genome Analysis

SARS-CoV-2 consensus sequences and variants were obtained from reads using the Medaka-based Artic-nCoV workflow v1.1.0 adapted by EPI2ME lab (rev. 41f235cdf1) [14]. Briefly, the reads were filtered by length to be between 400 and 700 bases long. They were then aligned on the Wuhan Hu-1 reference (RefSeq NC_045512.2) using minimap2 (PMID: 29750242). The variant calling step relied on Medaka [15].

## 3. Results

**Figure 1:**
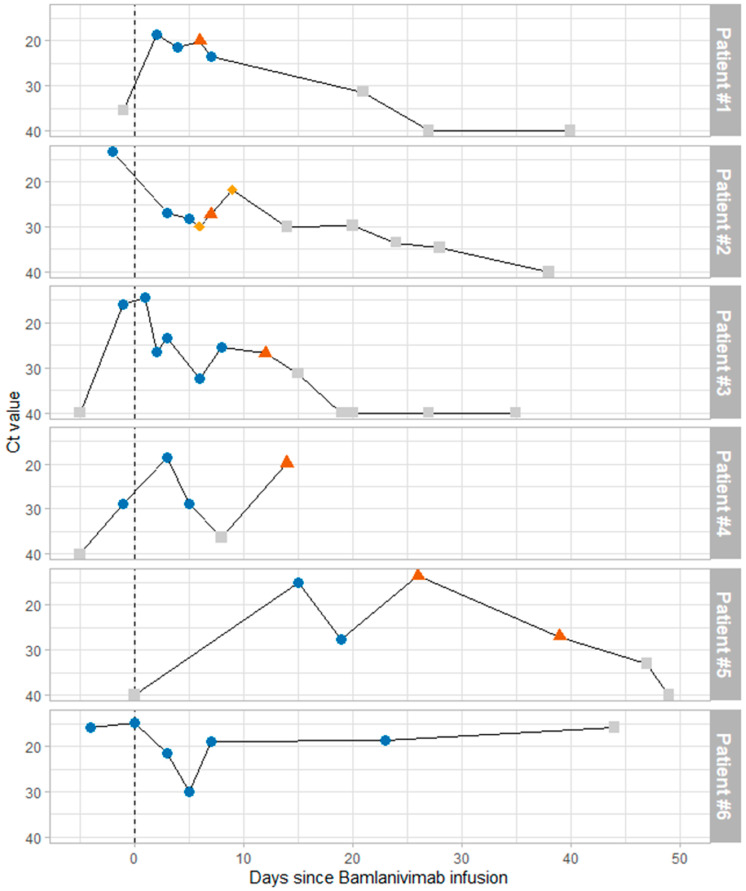
Virological evolution of patients after bamlanivimab infusion. Viral load, estimated by Ct values, is depicted on the vertical axis. Time since bamlanivimab infusion is depicted in days on the horizontal axis. Blue circles indicate that no mutation was detected at position 484 of the spike protein, red triangles indicate E484K mutation, and yellow diamonds indicate E484A mutation. Gray squares indicate the inability to check for E484 mutations due to low viral load (usually Ct values above 30).

During the two weeks we used bamlanivimab monotherapy in our center, six patients received a single dose of bamlanivimab. They were all males, with a median age of 65 years (range 35–97), and had more than three comorbidities among the following: diabetes, dialysis, hypertension, heart transplantation, COPD, stroke, and dementia. All the patients except Patient 2 were symptomatic, mostly with fever, and all the patients had a positive PCR for SARS-CoV-2 on the day of symptom onset or up to three days after. All were nosocomial SARS-CoV-2 infections. All patients were infected with a B.1.1.7 variant, which was the most frequent variant in France at the time, and no patients had E484 mutations at baseline. Bamlanivimab was infused in all patients within 4 days of symptom onset or the diagnosis of infection for asymptomatic patients (median 2 days). The viral evolution of each patient is depicted on Figure 1, and all mutations observed among the corresponding viral strains are depicted in Table 1 (spike gene) or Supplementary Table S1 (whole genome sequence).

Patient 1 was an 87-year-old male with a history of peripheral arterial obstructive disease, diabetes, hypertension, coronary heart disease, and grade III chronic renal disease. He was hospitalized for the management of diabetic foot ulcer and developed a fever four days after the admission. He tested positive for SARS-CoV-2 on the day of the fever and received bamlanivimab the day after. He did not need additional oxygen and had a favorable clinical evolution. The viral evolution presented an early selection of E484K mutation at day 6 after bamlanivimab infusion, detected by both specific RT-PCR screening and whole genome sequencing. At day 7, E484K was not detected by any of these tests, but a S494P, also described in several VOC, particularly under selection by immunoglobulins [16,17], was detected on the S gene. Such transient appearance of E484K has been observed in previous case reports [8,18]. After day 7, the viral load decreased regularly and was too low for repeating variant screening. The patient was transferred to a rehabilitation unit nine days after the infusion, and the viral load became negative at day 27 post-infusion. 

Patient 2 was a 35-year-old male with diabetes, hypertension, end-stage renal disease on dialysis, restrictive ventilatory disorder, and juvenile idiopathic arthritis. He was hospitalized for the initiation of hemodialysis after failure of peritoneal dialysis. Two weeks after admission he developed a fever and tested positive for SARS-CoV-2 on the same day. He received bamlanivimab two days after fever onset, did not need supplementary oxygen at any time, and had a favorable clinical evolution. The CT-scan four days after bamlanivimab therapy showed less than 15% lung opacification. Viral evolution showed the emergence of the E484A mutation at day 6 post-infusion and E484K at day 7. E484A was detected again at day 9. Concomitantly, the viral load increased from day 6 to day 9 but then decreased regularly before becoming negative at day 38. The patient stayed two months in the hospital because of a diabetic foot ulcer that existed before bamlanivimab infusion and was then discharged to a rehabilitation unit.

Patient 3 was a 61-year-old male, with a history of stroke, peripheral arterial obstructive disease, diabetes, hypertension, coronary heart disease, and end-stage renal disease on dialysis. He was hospitalized for the management of diabetic foot ulcers. One week after admission he developed a fever and was diagnosed with a nosocomial SARS-CoV-2 infection on the same day. He received bamlanivimab on the following day and had a CT-scan which showed a lung opacification of 30% of the parenchyma. Three days after the infusion of bamlanivimab he needed oxygen (3 L/min) and received steroids. Oxygen was weaned after four days. The viral evolution demonstrated a E484K selection 12 days after bamlanivimab infusion, quickly followed by viral load decrease, before becoming negative at day 18. The patient was still hospitalized two months later for various complications, including uncontrolled diabetes and acute coronary syndrome, both unrelated to COVID-19 or bamlanivimab.

Patient 4 was a 97-year-old male with dementia, hypertension, and diabetes. He was hospitalized for the management of diabetic foot ulcers. He developed fever and asthenia and needed oxygen (2 L/min) three weeks after admission. He tested positive for SARS-CoV-2 three days after symptom onset and received bamlanivimab the day after. He needed additional oxygen (4 L/min) two days after bamlanivimab infusion. He had a favorable clinical evolution and oxygen weaning after two days. Viral follow-up demonstrated a E484K selection on day 14, associated with a viral load increase. The patient was not sampled later as he developed necrotizing soft tissue infection, complicating the diabetic foot ulcer two weeks after bamlanivimab, and was transferred to palliative care. He died three weeks later of complications unrelated to COVID-19 or monoclonal therapy.

Patient 5 was a 64-year-old male with a history of diabetes, hypertension, and coronary heart disease, with heart transplantation five months before the episode. He was hospitalized following a stroke and tested positive for SARS-CoV-2 three weeks after admission, following contact with a COVID-19 patient. He received bamlanivimab one day after the diagnosis of SARS-CoV-2 infection. Eleven days after the administration of bamlanivimab, he needed supplementary oxygen (4 L/min). A CT-scan at the time showed lung opacification between 50 and 75%. He received corticosteroids for 10 days, and oxygen-weaning was possible 11 days later (day 24 after bamlanivimab). He presented a prolonged viral excretion, with positive viral loads up to day 47 after bamlanivimab infusion (day 36 after symptom onset). The viral load first decreased, from 15 to 27 Ct, before a new increase to 14 Ct on day 26 (day 15 after symptom onset), associated with the E484K detection at day 26 and 38. After day 26, the viral load slowly decreased and became negative at day 48.

Patient 6 was a 66-year-old male with a history of stroke, tuberculosis, diabetes, hypertension, rheumatoid arthritis, and end-stage renal disease. He had a kidney transplantation two months before the episode for which he was hospitalized. He tested positive for SARS-CoV-2 during a systematic screening and received bamlanivimab four days after the diagnosis of infection. He did not need supplementary oxygen and had a favorable clinical evolution, which allowed discharge from the hospital ten days later. He was concomitantly treated with belatacept for an acute graft rejection. Regarding viral follow-up, he presented a slight viral decrease from 14 Ct at bamlanivimab infusion to 30 Ct at day 5. At day 7, a new viral increase was observed, plateauing between 15 and 20 Ct up to 44 days after bamlanivimab infusion. No selection of the E484K was observed, neither by specific PCR screening nor by whole genome sequencing. Of note, a Q493R mutation, which may impact ACE-2 affinity [19], was observed in the S gene at day 23. Forty days after the administration of bamlanivimab, the patient was re-admitted with diarrhea and dyspnea. A CT-Scan found a COVID-19 pneumonia, with 40–50% of lung opacification. Despite steroids and tocilizumab, clinical condition worsened and the patient died a few days later, despite high-flow oxygen therapy.

## 4. Discussion

In this work, we describe the selection of E484K mutations among five out of six patients at high risk of severe COVID-19 who were treated with bamlanivimab monotherapy. Since the beginning of this study, the EUAs for bamlanivimab monotherapy have been revoked in the USA and in Europe due to the emergence of variants and the availability of combinations of monoclonal antibodies. This is supported by these results, which show a rate of resistance selection higher than described in early clinical trials (6 to 10%) [2]. All patients in this study were infected by the B.1.1.7 variant, which could increase the risk of E484A/K selection, as observed in the unique previous case report of bamlanivimab use on B.1.1.7 [7].

All six patients were at very high risk of severe COVID-19 and treated very early after symptom onset (between 0 and 4 days). Among them, two patients did not require oxygen, two required low-flow oxygen with a favorable outcome, one required oxygen for a few days and died from unrelated complications 5 weeks later, and one patient treated with belatacept for an acute kidney graft rejection had an initial favorable outcome but was hospitalized for a late worsening and died more than 6 weeks after administration of bamlanivimab (Patient 6).

Virologically, three patients cleared their nasopharyngeal viral shedding within one month after infusion, while two presented prolonged viral excretions for more than 40 days. Among these two, one presented a slow decrease from day 26 until becoming negative at day 48, while the last one (Patient 6) presented a highly stable viral load plateau associated with a clinical worsening, leading to death. Several factors could be at play to explain the variability in viral load kinetics, but their respective parts are unknown. In a recent work, nasopharyngeal viral load was linked to disease severity [20], as illustrated by Patient 6, who presented the longest viral excretion in our case series and died from COVID-19. Bamlanivimab administration and the development of resistance mutations could also impact viral fitness and kinetics. Moreover, differences in comorbidities and delays in bamlanivimab administration between patients could partially explain the observed differences.

This study confirms the clinical and virological findings from another observational case series in Germany, which also found that five of six immunocompromised patients treated with bamlanivimab monotherapy developed an E484K resistance mutation [21]. The very high level of E484K selection (5/6 patients) and other mutations observed emphasizes the importance of using combinations of monoclonal antibodies. It also highlights the need to carefully monitor resistance selection under monoclonal antibody therapy, especially with large circulation of the B.1.1.7 lineage. The impact of mutation E484A, observed for Patient 2, remains to be explored. Of note, E484K and E484A mutations correspond to two separate nucleotide changes (i.e., GAA to AAA for E484K and GAA to GCA for E484A). This suggests that for Patient 2, even if we were only able to detect one of these two viral populations at each time point, a switch occurred between minority viral subpopulations, rather than a continuous evolution of a single lineage. In the same way, a rapid E484K disappearance was also observed for Patient 1, as in two previous report cases without bamlanivimab [8,18]. The mechanisms and determinants explaining the maintenance or disappearance of viral mutations or the switches between resistance mutations, such as between E484K and E484A in Patient 2, remain to be explored and understood. Finally, all six patients presented a mutation associated with resistance or suspicion of resistance in the Spike protein, including E484A/K, but also S494P [16,17] and Q493R [19]. E484K and E484Q are rare without therapeutic antibody selective pressure, and several in vitro studies have reported that SARS-CoV-2 variants harboring these mutations are resistant to neutralization by the monoclonal antibody bamlanivimab [22,23]. Along with other case report series [21], the clinical observation of high rates of resistance emergence under such antibody monotherapy raises the concern of accelerating the E484K spread, along with other mutations, at a population level. A strict isolation of patients treated with monoclonal antibody monotherapy should therefore be recommended until the nasopharyngeal viral load is negative. Therapy with a cocktail of monoclonal antibodies reduces the risk of resistance but should be monitored closely. The individual clinical impact of resistance emergence during monoclonal antibody therapy has yet to be evaluated.

## Figures and Tables

**Table 1:** **Mutations observed on the S gene.** Each mutation from the Wuhan reference genome is indicated by a “•”.

Patient ID		#1				#2					#3				#4		#5			#6				
Days after Bamlanivimab Infusion		2	4	6	7	3	5	6	7	9	1	2	3	12	3	14	15	19	26	0	3	5	7	23
Ct Values		19	35	20	23	25	26	29	26	24	16	23	24	28	20	21	16	27	27	16	21	29	20	19
**Mutation**																								
**Nucleotide**	**Amino-acid**																							
*21736-C_T*	F58F	**•**	**•**	**•**	-	-	-	-	-	**•**	**•**	-	**•**	**•**	**•**	**•**	-	-	-	-	-	**•**	-	**•**
*21765-ATACATG_A*	Del 69-70	**•**	**•**	**•**	**•**	**•**	**•**	**•**	**•**	**•**	**•**	**•**	**•**	**•**	**•**	**•**	**•**	**•**	**•**	**•**	**•**	**•**	**•**	**•**
*21991-TTTA_T*	Del 144	**•**	**•**	**•**	**•**	**•**	**•**	**•**	**•**	**•**	**•**	**•**	**•**	**•**	**•**	**•**	**•**	**•**	**•**	**•**	**•**	**•**	**•**	**•**
***23012-G_A***	**E484K**	-	-	**•**	-	-	-	-	**•**	-	-	-	-	**•**	-	**•**	-	-	**•**	-	-	-	-	-
***23013-A_C***	**E484A**	-	-	-	-	-	-	•	-	**•**	-	-	-	-	-	-	-	-	-	-	-	-	-	-
*23040-A_G*	Q493R	-	-	-	-	-	-	-	-	-	-	-	-	-	-	-	-	-	-	-	-	-	-	**•**
*23042-T_C*	S494P	-	-	-	**•**	-	-	-	-	-	-	-	-	-	-	-	-	-	-	-	-	-	-	-
*23063-A_T*	N501Y	**•**	**•**	**•**	**•**	**•**	**•**	**•**	**•**	**•**	**•**	**•**	**•**	**•**	**•**	**•**	**•**	**•**	**•**	**•**	**•**	**•**	**•**	**•**
*23271-C_A*	A570D	**•**	**•**	**•**	**•**	**•**	**•**	**•**	**•**	**•**	**•**	**•**	**•**	**•**	**•**	**•**	**•**	**•**	**•**	**•**	**•**	**•**	**•**	**•**

## Data Availability

Data can be provided by the authors upon reasonable request.

## Supplementary Materials

The following are available online at https://www.mdpi.com/article/10.3390/v13081642/s1, Table S1: Mutations observed for each viral sequence. Each mutation from the Wuhan reference genome is indicated by a “•”.
