# Supplementary material for: Emergence of E484K Mutation Following Bamlanivimab Monotherapy among High-Risk Patients Infected with the Alpha Variant of SARS-CoV-2
Source: Viruses. 2021 Aug 19;13(8):1642. doi: 10.3390/v13081642 (PMC8402761; doi:10.3390/v13081642)
Supplement: Supplementary file 1 [file viruses-13-01642-s001.zip › viruses-1330783-SI.pdf]

Supplementary Table S1. Mutations observed for each viral sequence. Each mutation from the Wuhan reference genome is indicated by a "•".

Patient ID			#1				#2					#3				#4		#5			#6				
**Days after Bamlanivimab infusion**			**2**	**4**	**6**	**7**	**3**	**5**	**6**	**7**	**9**	**1**	**2**	**3**	**12**	**3**	**14**	**15**	**19**	**26**	**0**	**3**	**5**	**7**	**23**
**Ct values**			**19**	**35**	**20**	**23**	**25**	**26**	**29**	**26**	**24**	**16**	**23**	**24**	**28**	**20**	**21**	**16**	**27**	**27**	**16**	**21**	**29**	**20**	**19**
**Gene**	**Mutation**																								
	**Nucleotide**	**Amino-acid**																							
	913-C_T	S216S	•	•	•	•	•	•	•	•	•	•	•	•	•	•	•	•	•	•	•	•	•	•	•
	1489-C_T	G408G	•	•	•	•	•	-	•	•	•	•	•	•	•	•	•	-	-	-	•	•	•	•	•
	1730-G_A	E489K	•	•	•	•	•	•	•	•	•	•	•	•	•	•	•	-	-	-	•	•	•	•	•
	2110-C_T	N615N	•	•	•	•	•	•	•	•	•	•	•	•	•	•	•	-	-	-	•	•	•	•	•
	3037-C_T	F924F	•	•	•	•	-	-	•	•	•	•	•	•	•	•	•	•	•	•	•	•	•	-	•
	3267-C_T	T1001I	•	•	•	•	•	•	•	•	•	•	•	•	•	•	•	•	•	•	•	•	•	•	•
	3773-C_T	R1170C	-	-	-	-	-	-	•	-	•	-	-	-	-	-	-	-	-	-	-	-	-	-	-
	5388-C_A	A1708D	•	•	•	•	•	•	•	•	•	•	•	•	•	•	•	•	•	•	•	•	•	•	•
	5986-C_T	F1907F	•	•	•	•	•	•	•	•	•	•	•	•	•	•	•	•	•	•	•	•	•	•	•
	6954-T_C	I2230T	•	•	•	•	•	•	•	•	•	•	•	•	•	•	•	•	•	•	•	•	•	•	•
	7184-A_G	I2307V	•	-	•	•	•	•	•	•	•	•	-	-	•	•	•	-	-	-	-	-	•	•	-
	11288-GTCTGGTTTT_G	Del 3675-3677	•	•	•	•	•	•	•	•	•	•	•	•	•	•	•	•	•	•	•	•	•	•	•
	11565-C_T	P3767L	•	•	•	•	•	•	•	•	•	•	•	•	•	•	•	-	-	-	•	•	•	•	•
	12895-A_C	E4210D	-	-	-	-	-	-	-	•	-	-	-	-	-	-	-	-	-	-	-	-	-	-	-
ORF1b	*13899-T_G*	D144E	-	-	-	-	-	-	-	-	-	-	-	-	-	-	-	•	•	•	-	-	-	-	-
	14120-C_T	P218L	•	•	•	•	•	•	•	•	•	•	•	•	•	•	•	-	-	-	•	•	•	•	•
	14277-G_A	R270R	-	-	-	-	-	-	-	-	-	-	-	-	-	-	-	•	•	•	-	-	-	-	-
	14408-C_T	P314L	•	•	•	•	•	•	•	•	•	•	•	•	•	•	•	•	•	•	•	•	•	•	•
	14676-C_T	P403P	•	•	•	•	•	•	•	•	•	•	•	•	•	•	•	•	•	•	•	•	•	•	•
	15096-T_C	N543N	-	-	-	-	-	-	-	-	-	-	-	-	-	-	-	•	•	•	-	-	-	-	-
	15279-C_T	H604H	•	•	•	•	•	•	•	•	•	•	•	•	•	•	•	•	•	•	•	•	•	•	•
	15672-G_T	E735D	-	-	-	-	-	-	-	-	-	-	-	-	-	-	-	•	•	•	-	-	-	-	-
	16176-T_C	T903T	•	•	•	•	•	•	•	•	•	•	•	•	•	•	•	•	•	•	•	•	•	•	•
	16254-T_C	V929V	-	-	-	-	-	-	-	-	-	-	-	-	-	-	-	-	-	•	-	-	-	-	-
S	*21736-C_T*	F58F	•	•	•	-	-	-	-	-	•	•	-	•	•	•	•	-	-	-	-	-	•	-	•
	21765-ATACATG_A	Del 69-70	•	•	•	•	•	•	•	•	•	•	•	•	•	•	•	•	•	•	•	•	•	•	•
	21991-TTTA_T	Del 144	•	•	•	•	•	•	•	•	•	•	•	•	•	•	•	•	•	•	•	•	•	•	•
	23012-G_A	**E484K**	-	-	•	-	-	-	-	•	-	-	-	-	•	-	•	-	-	•	-	-	-	-	-
	23013-A_C	**E484A**	-	-	-	-	-	-	•	-	•	-	-	-	-	-	-	-	-	-	-	-	-	-	-
	23040-A_G	Q493R	-	-	-	-	-	-	-	-	-	-	-	-	-	-	-	-	-	-	-	-	-	-	•
	23042-T_C	S494P	-	-	-	•	-	-	-	-	-	-	-	-	-	-	-	-	-	-	-	-	-	-	-
	23063-A_T	N501Y	•	•	•	•	•	•	•	•	•	•	•	•	•	•	•	•	•	•	•	•	•	•	•
	23271-C_A	A570D	•	•	•	•	•	•	•	•	•	•	•	•	•	•	•	•	•	•	•	•	•	•	•
	23403-A_G	D614G	•	•	•	•	•	•	•	•	•	•	•	•	•	•	•	•	•	•	•	•	•	•	•
	23604-C_A	P681H	•	•	•	•	•	•	•	•	•	•	•	•	•	•	•	•	•	•	•	•	•	•	•
	23709-C_T	T716I	•	•	•	•	•	•	•	•	•	•	•	•	•	•	•	•	•	•	•	•	•	•	•
	24328-G_C	L922F	-	-	-	-	-	-	-	-	-	-	-	-	•	-	-	-	-	-	-	-	-	-	-
	24506-T_G	S982A	•	•	•	•	•	•	•	•	•	•	•	•	•	•	•	•	•	•	•	•	•	•	•
	24914-G_C	D1118H	•	•	•	•	•	•	•	•	•	•	•	•	•	•	•	•	•	•	•	•	•	•	•
	24919-C_T	N1119N	-	-	-	-	-	-	-	-	-	-	-	-	-	-	-	•	•	•	-	-	-	-	-
	25352-G_T	V1264L	-	-	-	-	-	-	-	-	-	-	-	-	-	-	-	•	•	•	-	-	-	-	-
ORF3a	*25785-G_T*	W131C	-	-	-	-	-	-	-	-	-	-	-	-	-	-	-	•	•	•	-	-	-	-	-
ORF7a	*27647-A_G*	K85R	•	-	-	-	-	-	-	-	-	-	-	-	-	-	-	-	-	-	-	-	-	-	-
ORF8	*27972-C_T*	Q27*	•	•	•	•	•	•	•	•	•	•	•	•	•	•	•	•	•	•	•	•	•	•	•
	28048-G_T	R52I	•	•	•	•	•	•	•	•	•	•	•	•	•	•	•	•	•	•	•	•	•	•	•
	28095-A_T	K68*	•	•	•	•	•	•	•	•	•	•	•	•	•	•	•	•	•	•	•	•	•	•	•

	28111-A_G	Y73C	•	•	•	•	•	•	•	•	•	•	•	•	•	•	•	•	•	•	•	•	•	•	•
None	*28270-TA_T*	Non-coding	•	•	•	•	•	•	•	•	•	•	•	•	•	•	•	•	•	•	•	•	•	•	•
N	*28280-GAT_CTA*	D3L	•	•	•	•	•	•	•	•	•	•	•	•	•	•	•	•	•	•	•	•	•	•	•
	28881-GGG_AAC	Del 203-204	•	•	•	•	•	•	•	•	•	•	•	•	•	•	•	•	•	•	•	•	•	•	•
	28977-C_T	S235F	•	•	•	•	•	•	•	•	•	•	•	•	•	•	•	•	•	•	•	•	•	•	•
None	*29751-G_A*	Non-coding	-	-	-	-	-	-	-	-	-	-	-	-	-	-	-	•	•	•	-	-	-	-	-
